# Isolation, characterization, and genomic analysis of a novel phage WSPA with lytic activity against *Serratia marcescens*

**DOI:** 10.1128/spectrum.02716-24

**Published:** 2025-08-05

**Authors:** Lijuan Li, Kunkun Li, Yu Mi, Xiumei Wu, Youhong Zhong, Chenggui Zhang, Peng Wang, Hairong Zhao

**Affiliations:** 1 Yunnan Provincial Key Laboratory of Entomological Biopharmaceutical R&D, College of Pharmacy, Dali University 66359, Dali, Yunnan, China; 2 The First Affiliated Hospital of Dali University 687235 , Dali, Yunnan, China; 3 National-Local Joint Engineering Research Center of Entomoceutics, Dali, China; 4 Yunnan Institute for Endemic Disease Control and Prevention 718973, Dali, China; University of Pittsburgh School of Medicine, Pittsburgh, Pennsylvania, USA

**Keywords:** *Serratia marcescens*, *Periplaneta americana *L., bacteriophage therapy, multidrug resistance, environmental microbiology

## Abstract

*Serratia marcescens* is an established pathogen implicated in hospital-acquired infections and is notorious for its propensity to form biofilms on medical devices, leading to persistent environmental contamination and increased infection risks. Addressing this challenge, our study introduces a novel bacteriophage, Weishan phage (WSPA), isolated from the gut of *Periplaneta americana* L., exhibiting potent activity against multidrug-resistant strains of *S. marcescens*. Through comprehensive genomic and proteomic characterization, we classified phage WSPA as a member of the genus *Muldoonvirus* within the class *Caudoviricetes*. The WSPA phage has a double-stranded DNA genome of 173,655 base pairs and a GC content of 40.09%. Of the 273 open reading frames identified, 124 encode for proteins with recognized functions in the National Center for Biotechnology Information (NCBI) database, while the remaining 149 are of unknown function. Additionally, we identified six tRNA genes and did not identify any virulence or antibiotic resistance genes. However, given the presence of numerous hypothetical genes with unknown functions, this phage may possess certain therapeutic safety potential, though additional research validation is still required. Comparative genomic analysis revealed that WSPA shares 86.92% sequence identity with the known *Serratia phage 4S* (MW082584.1). We further assessed the phage’s one-step growth characteristics, thermal and pH stability, and determined its host range, which are critical for its application in environmental and clinical settings. Our findings suggest that bacteriophage WSPA could serve as an eco-friendly and effective agent in controlling *S. marcescens* infections, with promising implications for phage therapy and biocontrol in healthcare environments.

**IMPORTANCE:**

This study isolated a novel phage, WSPA, from *Periplaneta americana* L. gut that specifically targets multidrug-resistant *Serratia marcescens*. Genomic analysis identified WSPA as a new Muldoonvirus member lacking virulence/resistance genes. With excellent stability and lytic activity, WSPA shows potential for hospital infection control. As the first phage isolated from the cockroach gut, this work expands phage resources and supports medicinal insect phage library development, advancing phage therapy and biocontrol applications.

## INTRODUCTION

*Serratia marcescens* is a Gram-negative opportunistic pathogen within the *Enterobacteriaceae* family (1). It is characterized by its unique biological features, such as its motility conferred by peritrichous flagella, absence of a capsule and endospores, and resistance to multiple antibiotics (2). *S. marcescens* is widely distributed in nature, inhabiting soil, water bodies, plants, and animals, including the human urinary and respiratory tracts (3). While typically harmless to healthy individuals, it can cause serious infections in immunocompromised hosts (4), including pneumonia (5), sepsis, and meningitis (6, 7). Nosocomial infections are primarily caused by pathogens present in healthcare environments, among which certain strains of *S. marcescens* have emerged as clinically significant pathogens due to their multidrug-resistant properties. This resistance complicates treatment and heightens the challenge of infection control (3, 8). Moreover, the environmental tolerance of *S. marcescens* allows it to survive in the hospital environment, increasing the risk of cross-infection and posing threats to water quality and soil ecosystems (9, 10).

Antibiotic therapy remains the main approach to treating infections caused by *S. marcescens* (3). However, the extensive use of antibiotics has led to a high level of multidrug resistance in *S. marcescens*(11, 12) , reducing treatment efficacy and increasing medical costs and patient risks. At the same time, the misuse of antibiotics disrupts microbial balance (13), promoting the spread of resistant bacteria in the environment and causing public health issues (14). Bacteriophage therapy offers a promising alternative, as it can target and inactivate specific pathogens (15, 16) without directly affecting other microorganisms (17–20). This approach can address bacterial resistance development while reducing the risk of side effects.

*Periplaneta americana* L. (*P. americana*), belonging to the order *Blattodea* under the class *Insecta*, has an evolutionary history exceeding 300 million years (21). While recognized as a potential disease vector, it holds medicinal value in traditional Chinese medicine. The Compendium of Materia Medica documents its efficacy in activating blood circulation to resolve blood stasis, detoxifying, reducing swelling, and promoting diuresis. Modern research has identified additional properties, including antitumor (22), antiviral (23), antioxidant (24), anti-inflammatory, and analgesic effects (25). The commercially available "Kangfuxin Liquid" is a traditional Chinese medicine preparation derived from ethanol extracts of dried *P. americana*. Studies have demonstrated that this formulation exhibits therapeutic effects by modulating epidermal growth factors and promoting tissue repair (26). Yi Chen et al. demonstrated in a rat model that *P. americana* extract improves hepatic function and fibrosis indices, while effectively attenuating liver fibrosis progression (27).

Currently, bacteriophages are mostly isolated from environments such as soil or sewage (28). Reports on the extraction and isolation of bacteriophages from insects are absent, and research into discovering and developing medicinal phages from insects is still blank. Given the potential medicinal value of *P. americana*, along with its strong survival ability and other biological characteristics, the intestinal microbial community of *P. americana* may harbor phages that target *S. marcescens*, providing a new way to obtain phage resources.

This study analyzes the richness of bacteriophage in the gut of *P. americana* through metagenomic sequencing technology, providing a novel approach to researching the microecology of pathogenic bacteriophage in the insect gut. It lays the foundation for establishing a medicinal insect bacteriophage library. This research investigates the biological and genomic characteristics of an *S. marcescens* phage isolated from *P. americana*, analyzing basic characteristics and environmental tolerance of the *S. marcescens* phage. This research provides a theoretical basis and technical guidance for preventing and controlling *S. marcescens* infections in hospitals and for developing *S. marcescens* phage biocontrol preparations.

## MATERIALS AND METHODS

### Bacterial strains and growth conditions

This study used a total of 47 test bacterial strains (Table 1), including 12 strains of *Shigella*, 6 strains of *Yersinia pseudotuberculosis*, and 2 strains of *Yersinia enterocolitica*, all sourced from the Key Laboratory of Natural Focus Disease Prevention and Control Technology in Yunnan Province. Additionally, 17 strains of *S. marcescens* and 10 other clinical bacterial strains were obtained from the People’s Hospital of Dali Prefecture. The strain designated for phage isolation in this study was *S. marcescens *18, provided by the People’s Hospital of Dali Prefecture. The original bacterial strains were preserved at −80°C. All bacteria were cultured in Luria-Bertani (LB) broth supplemented with 0.9% (wt/vol) NaCl at 37°C.

**TABLE 1:** The 47 bacterial strains used in this study belong to the following genera^*a*^

Strain category	Strain name
*Serratia marcescens*	40, 43, 42, 41, *Serratia odorifera*, 18=Ref, 20, 21, 29, 7, 45, 46, 47, 26, 44, 13, 14
*Shigella*	*Shigella flexneri *1a,* Shigella flexneri *2a,* Shigella flexneri *3a,* Shigella flexneri *4a,* Shigella flexneri *IV,* Shigella flexneri *serotype X,* Shigella sonnei *I,* Shigella sonnei *II,* Shigella dysenteriae *I,* Shigella dysenteriae *II,* Shigella boydii, Shigella sonnei*
*Yersinia pseudotuberculosis*	PSTI, PSTII, PSTIII, PSTIV, PSTV, PSTVI
*Yersinia enterocolitica*	52202, 52301
Other clinical strains	*Pseudomonas aeruginosa, Klebsiella pneumoniae, Escherichia, Acinetobacter baumannii, Escherichia, Salmonella typhi, Enterobacter cloacae, Buttiauxella agrestis, *ATCC25922,* Staphylococcus epidermidis*

^*a*^ "Ref" indicates that the bacterial strain was used as a host strain in the table.

### Extraction of the intestine from *Periplaneta americana*

The *P. americana* specimens used in this study were obtained from a standardized breeding facility in Weishan County, Dali Bai Autonomous Prefecture, Yunnan Province, China. Prior to experimentation, the specimens were cold-anesthetized at 4°C for 15 minutes until complete immobilization was achieved. Subsequent procedures were conducted in a biosafety cabinet, where the specimens were first surface-sterilized with 75% ethanol, then dissected along the ventral midline using sterile ophthalmic scissors and micro-forceps to isolate intact intestinal tissues. Transfer the intestine into a sterile centrifuge tube containing SM buffer (Biosharp, BL1199B, China). To acquire comprehensive data sets on host microbiota and their associated phages, we analyzed the quantitative relationships between hosts and phages to identify the dominant phage populations in the intestinal tract of *P. americana* for targeted research. Following standardized preprocessing, intestinal content samples from *P. americana* were submitted to LingEn Biotech Co., Ltd., for metagenomic sequencing analysis.

### DNA extraction, library preparation, and sequencing of the intestine of *Periplaneta americana*

Microbial DNA was extracted from the intestines of *P. americana* using the E.Z.N.A. stool DNA Kit (Omega Bio-Tek, Norcross, GA, USA) according to the manufacturer’s protocols. Metagenomic shotgun sequencing libraries were constructed and sequenced at Shanghai Biozeron Biological Technology Co., Ltd. In brief, for each sample, 1 µg of genomic DNA was sheared by the Covaris S220 Focused-ultrasonicator (Woburn, MA, USA), and sequencing libraries were prepared with a fragment length of approximately 450 bp. All samples were sequenced in the Illumina HiSeq X instrument with pair-end 150 bp (PE150) mode. Raw sequence reads underwent quality trimming using (http://www.usadellab.org/cms/uploads/supplementary/Trimmomatic) Trimmomatic (ILLUMINACLIP:adapters.fa:2:30:10 SLIDINGWINDOW:4:15 MINLEN:75) to remove adapter contaminants and low-quality reads (29). Reads through quality control were then mapped against both the human genome (Version: hg19) and the *P. americana* genome (https://www.ncbi.nlm.nih.gov/genome/?term=txid6978 [Organism:noexp]) by the BWA-MEM algorithm (http://bio-bwa.sourceforge.net/bwa.shtml, parameters: -M -k 32 -t 16). The reads removing human genome contaminations, host-genome contaminations, and low-quality data were called clean reads and used for further analysis.

### Reads-based taxonomic annotation

Taxonomy of clean reads for each sample was determined by Kraken2 (Version: 2.0.6 --threads 16 --quick --report-zero-counts --gzip-compressed --paired) (30) using a customized Kraken database. The customized kraken database included all bacteria, archaea, fungi, virus, protozoa, and algae genome sequences in the National Center for Biotechnology Information (NCBI) RefSeq database (release number: 90). All reads were classified to seven phylogenetic levels (domain, phylum, class, order, family, genus, species) or unclassified. The abundances of taxonomy were estimated by Bracken (https://ccb.jhu.edu/software/bracken/), which can produce accurate species- and genus-level abundance even in multiple near-identical species. In this study, the relative abundance at a given taxonomic level (e.g., phylum, class, genus) refers to the sum of abundance values from all species belonging to that level.

### Metagenomic *de novo* assembly, gene prediction, and annotation

Clean sequence reads were assembled into a set of contigs for each sample using MegaHit (Version: 1.1.1-2-g02102e1) with “--min-contig-len 500” parameters (31). The open reading frames (ORFs) of assembled contigs were predicted using Prodigal (Version:2.6.3) (32), and all ORFs were generated to a set of unique genes after clustering using CD-HIT (version:4.8.1, parameters: -n 9 -c 0.95 -G 0 -M 0 -d 0 -aS 0.9 -r 1) (33). The longest sequence of each cluster was considered the representative sequence of each gene in the unique-gene set. In order to calculate the gene abundance within total samples, Salmon software (https://github.com/COMBINE-lab/salmon) (34) was applied to get the reads number for each gene. Finally, the gene abundance was calculated using the following formulas:


Ab(S)=Ab(U)+Ab(M),

$$Ab(U)=\sum_{L=1}^{M}1/L,$$

$$Ab(M)=\sum_{L=1}^{M}Co\times 1/L,$$

$$Co=Ab(U)/\sum_{L=1}^{N}Ab(U_{L}).$$

Ab(S), gene abundance; Ab(U), single-mapping reads abundance; Ab(M), multi-mapping reads abundance; and L, length of gene sequence (35). The gene set was compared with the non-redundant (NR) database using BLASTP (https://blast.ncbi.nlm.nih. gov/Blast.cgi). Species annotations were obtained through the corresponding taxonomy information database of the NR library. The abundance of each species was calculated by summing the gene abundances corresponding to that species. The abundance of species was then statistically analyzed at various taxonomic levels (Domain, Kingdom, Phylum, Class, Order, Family, Genus, Species) for each sample, thereby constructing the abundance profile at the corresponding taxonomic levels.

### Host bacterial preparation

The cryopreserved *S. marcescens* 18 bead stock was removed from −80°C storage and streaked densely onto LB solid medium (Table 2), then incubated inverted at 37°C for 18–24 hours. A single colony was inoculated into LB liquid medium (5 mL) and cultured at 37°C with shaking at 220 rpm for 8–10 hours to reach log phase (OD600 ≈ 0.6–0.7).

**TABLE 2:** Preparation of LB medium (400 mL as an example)

Component (g)	Solid medium	Semi-solid medium	Liquid medium
Agar	6	1.6	–^*a*^
Tryptone (1.0%)	4	4	4
Yeast extract (0.5%)	2	2	2
Na_2_HPO_4_ (0.25%)	1	1	1
NaCl (0.9%)	3.6	3.6	3.6

^*a*^ The “–” indicates that no amount of agar was added to the LB liquid medium.

### Phage isolation

The samples of *P. americana* were grouped in tens, crushed, and placed into centrifuge tubes. Peptone sorbitol bile broth (Sigma-Aldrich, Germany) was added to the tubes, and the mixture was designated as WSPA. WSPA (5 mL) was filtered through a 0.22 µm membrane and mixed with LB liquid medium, SM buffer, and the host bacterium *S. marcescens *18. The mixture was then placed in a constant-temperature air bath shaker at 220 rpm and 37°C for overnight incubation. The clarity of the liquid in the centrifuge tube was observed to determine whether it changed from turbid to clear, providing a preliminary indication of the presence of phages in the sample. The double-layer agar (DLA) method was employed for detection: the overnight-amplified WSPA sample was filtered through a 0.22 µm membrane, and the entire filtrate was mixed with the host bacterial *S. marcescens *18 suspension (100 µL). The mixture was then gently poured onto a 9 cm LB solid medium plate and incubated overnight at 37°C to identify phage plaques.

### Phage purification

Phage plaques were placed into a flask containing LB broth medium mixed with the host bacterium *S. marcescens* 18 and incubated overnight at 37°C with shaking at 220 rpm in a constant temperature shaker incubator (INFORSAF CH-4103, Sweden). The mixture was then filtered through a 0.22 µm membrane and plated. This process was repeated 5 to 6 times. During the purification process, it was found that the optimal plaque formation time for this phage was 6 hours, and the optimal culture temperature was 37°C. All subsequent experiments were conducted using these optimal culture time and temperature conditions.

### Titer determination

Ratio dilution (RD) method was used for titer determination by DLA. Initially, 900 µL of LB liquid medium and 100 µL of proliferated phage filtrate were added to a sterile centrifuge tube. Sequentially, a 10-fold serial dilution was performed to achieve the required multiplicity. From each dilution, 10 µL were mixed with 100 µL of the host bacterium *S. marcescens* 18. The mixture was then incubated at 37°C for 6–8 hours. Phage plaques were counted, with three parallel plates prepared for each dilution. The average value was calculated to determine the phage potency.

### Electron microscopy observation

Phages were amplified to a titer of 1 × 10^8^ PFU/mL, then filtered and stored at 4°C. Following the research by Sambrook et al. (36), CsCl gradient density centrifugation was used to separate and purify the phages. The phage solution (20 µM, 1 × 10^8^ PFU/mL) was applied evenly onto carbon-coated copper grid slides (AGS160-3H, Agar Scientific) for adsorption (10 minutes). The phages were then stained with a 2% (wt/vol) solution of phosphotungstic acid (pH 7.2). Electron microscopy observations were conducted using a Tecnai 12 transmission electron microscope (Philips Electron Optics, Eindhoven, The Netherlands).

### Host range

Host range analysis was performed using 47 test strains from five species (Table 1). The bacterial strains for host range analysis were streaked onto LB solid medium, cultured for 18–24 hours, and stored at 4°C for later use. The experiment was conducted at three cultivation temperatures (21°C, 28°C, and 37°C) with three replicates for each condition. For each bacterial strain under different temperatures, phage lysate (5 µL) was added, followed by 6 hours of incubation to examine plaque formation and determine the phage’s lytic activity against each host strain.

### Multiplicity of infection and one-step growth curve

The phage and its host bacterium *S. marcescens *18 were amplified to achieve a phage titer of 1 × 10⁸ PFU/mL and a bacterial concentration of 1 × 10⁷ CFU/mL. The bacterial suspension was homogenized and its concentration standardized. The phage lysate was serially diluted 10-fold, then mixed with the bacterial culture at multiplicity of infection (MOI) ratios of 0.00001, 0.0001, 0.01, 0.1, 1, and 10 (calculated as phage titer/host bacterial concentration). The mixtures were incubated at 37°C with continuous shaking (220 rpm) for 6 hours in a constant-temperature incubator shaker (INFORSAF CH-4103, Sweden), followed by filtration through a 0.22 µm sterile membrane filter. Subsequently, the host bacteria (100 µL) were mixed with phage filtrate (10 µL) at each corresponding MOI and allowed to stand for 10 minutes. The phage titer was then determined using the DLA plaque assay. This entire procedure was repeated in triplicate to ensure experimental reproducibility.

The phage-to-host bacteria ratio was adjusted according to the MOI. Aliquots (500 µL each) of the phage suspension and bacterial suspension, optimized to the appropriate MOI, were thoroughly mixed and then agitated at 37°C and 220 rpm for 10 minutes in a constant-temperature incubator shaker. The mixture was then centrifuged using a high-speed refrigerated centrifuge (Merck & Co., Inc., USA) at 4°C (12,000 rpm, 3 minutes), washed three times, and resuspended in pre-warmed LB liquid medium. Samples were taken every 5 minutes up to 20 minutes and then every 10 minutes up to 90 minutes. The phage titer was determined using the DLA method. A one-step growth curve was plotted, and the latency period and rise period of phage WSPA were calculated using the research method of Pujato et al. (37).

### Stability analysis

To observe the effects of various temperatures and pH values on the phage, the phage with an initial titer of 1 × 10⁸ PFU/mL was incubated at various temperatures (4°C, 21°C, 37°C, 50°C, 60°C, and 70°C) and across a pH range of 0 to 14 for 1 hour. Using the DLA method, the host bacterium *S. marcescens *18 (100 μL) was mixed with the phage diluted to approximately 1 × 10^6^ via the RD method. The titer was measured using the spot assay method. After incubation under different conditions, the phage was further incubated at 37°C for 6 hours, and the plaques were counted. The experiment was repeated three times, and the average values were calculated. The pH values were adjusted using PBS buffer with HCl or NaOH and remained stable during the 1 hour incubation at 37°C. The base solution used was LB liquid medium, which served to dilute the phage and culture the bacteria.

### Extraction of the phage WSPA genome

The genome of phage WSPA was extracted using a λ-phage nucleic acid extraction kit (ABigen, China) following the manufacturer’s instructions. In a preheated water bath at 37°C, DNase I and RNase A were added to the phage, and after 30 minutes, EDTA (20 mM) and NaCl (2.4 g) were added. After dissolving by shaking, the mixture was placed in an ice bath for 1 hour. The solution was then centrifuged at 4°C (7,000 rpm, 10 minutes), and 40 mL of the supernatant was collected. PEG 8000 was added to a final concentration of 10%, and the mixture was shaken and left overnight in an ice bath to precipitate. After centrifugation at 4°C (12,000 rpm, 45 minutes), the supernatant was discarded, and the pellet was inverted onto absorbent paper to collect the phage pellet. The phage pellet was resuspended in lysis buffer and incubated at 70°C. Following this, it was mixed with 20% SDS, cooled, and mixed with an impurity precipitation liquid before centrifugation at 4°C (12,000 rpm, 10 minutes). The supernatant was added to LB liquid medium and incubated at 37°C, after which it was passed through an activated carbon adsorption column multiple times. The column was washed with a wash buffer containing anhydrous ethanol. After centrifugation at 4°C (12,000 × *g*, 1 minute) to remove the wash buffer, the DNA was eluted with preheated elution buffer. After centrifugation (12,000 × *g*, 1 minute) to collect the eluate, the DNA was stored at −80°C.

### Phage WSPA sequencing analysis and functional annotation

The DNA that passed the preliminary quality check was provided to LingEn Biotech in Shanghai, China. The genomic DNA was quantified using a TBS-380 fluorometer (Turner BioSystems Inc., Sunnyvale, California). High-quality DNA samples (OD_260/280_ = 1.8–2.0, >6 µg) were used to construct the fragment library. A DNA library with a size range of 300–500 bp was constructed using the Illumina TruSeq Nano DNA Sample Prep Kit, followed by 300 bp paired-end sequencing. Raw paired-end reads were trimmed and quality-controlled using Trimmomatic (Version 0.36, cms/uploads/supplementary/Trimmomatic) with parameters (SLIDINGWINDOW:4:15 MINLEN:75). The clean data obtained through the above quality control process were used for further analysis.

We performed genome assembly using ABySS (http://www.bcgsc.ca/platform/bioinfo/software/abyss) with multiple k-mer parameters and obtained the optimal assembly results. The gene model of the assembled WSPA strain genome was constructed using *de novo* prediction methods. ORF identification was performed using GeneMark software. Functional annotation was accomplished by comparing the translated ORF products against the NCBI NR database, SwissProt (http://uniprot.org), Kyoto Encyclopedia of Genes and Genomes (KEGG) (http://www.genome.jp/kegg/), and Clusters of Orthologous Genes (COG) (http://www.ncbi.nlm.nih.gov/COG) databases using the blastp module (e-value cutoff < 10^−5^) (38). The closest protein homologs for coding sequences (CDSs) were identified using QIAGEN CLC Genomics Workbench 24.0.2 software, and a genomic circular map was generated using the CGView online tool (https://proksee.ca/). Additionally, tRNAscan-SE (v1.23, http://lowelab.ucsc.edu/tRNAscan-SE) was used to identify tRNAs, and RNAmmer (v1.2, http://www.cbs.dtu.dk/services/RNAmmer/) was used to determine rRNAs. The Comprehensive Antibiotic Resistance Database (https://card.mcmaster.ca/) and the Virulence Factor Database (http://www.mgc.ac.cn/VFs/main.htm) online platforms were used to compare and detect antibiotic resistance genes and virulence factors of the phage. The comparative genomic analysis of phage WSPA was performed using Easyfig (Version: 2.2.5) (39).

### Phylogenetic tree construction

BLASTp sequence identity analysis was performed in the NCBI database based on the terminase large subunit. The final selection included three Myovirus phages, three siphovirus phages, and five *Muldoonvirus* phages, with *Escherichia phage T7* serving as the outgroup. The highly conserved terminal large subunit coding sequences of these 12 phages were downloaded, with a BLASTp cutoff value of <1e^−5^. Based on the terminal large subunit sequences, multiple sequence alignment was performed using MEGA11 (Version: 11.0.13) (40). A phylogenetic tree was constructed using the neighbor-joining method and the Poisson correction model (41), with 1,000 bootstrap replicates to assess branch reliability. The tree is drawn to scale, with branch lengths in the same units as the evolutionary distances used to infer the phylogenetic tree. The evolutionary distances are expressed as the number of amino acid substitutions per site. This analysis involved 13 fragments of the terminal large subunit. For each sequence pair, all ambiguous positions were removed (pairwise deletion option). The final data set consisted of 740 positions. Evolutionary analyses were conducted in MEGA11 (42).

### Statistical analysis

Data were presented as mean values with standard deviations. Statistical parameters were calculated using GraphPad Prism 10 software (Version: 10.1.2). The significance among more than two groups was determined using one-way analysis of variance. All experiments were conducted blindly and independently repeated under the same conditions. Statistical significance was defined as a *P*-value of <0.05.

### Bioinformatics tools parameter usage statement

For all bioinformatics tools used in this study, specified parameters are documented in the text, while unspecified tools were applied with default parameters.

## RESULTS

### Phylogenetic annotation based on reads

**Fig 1:**
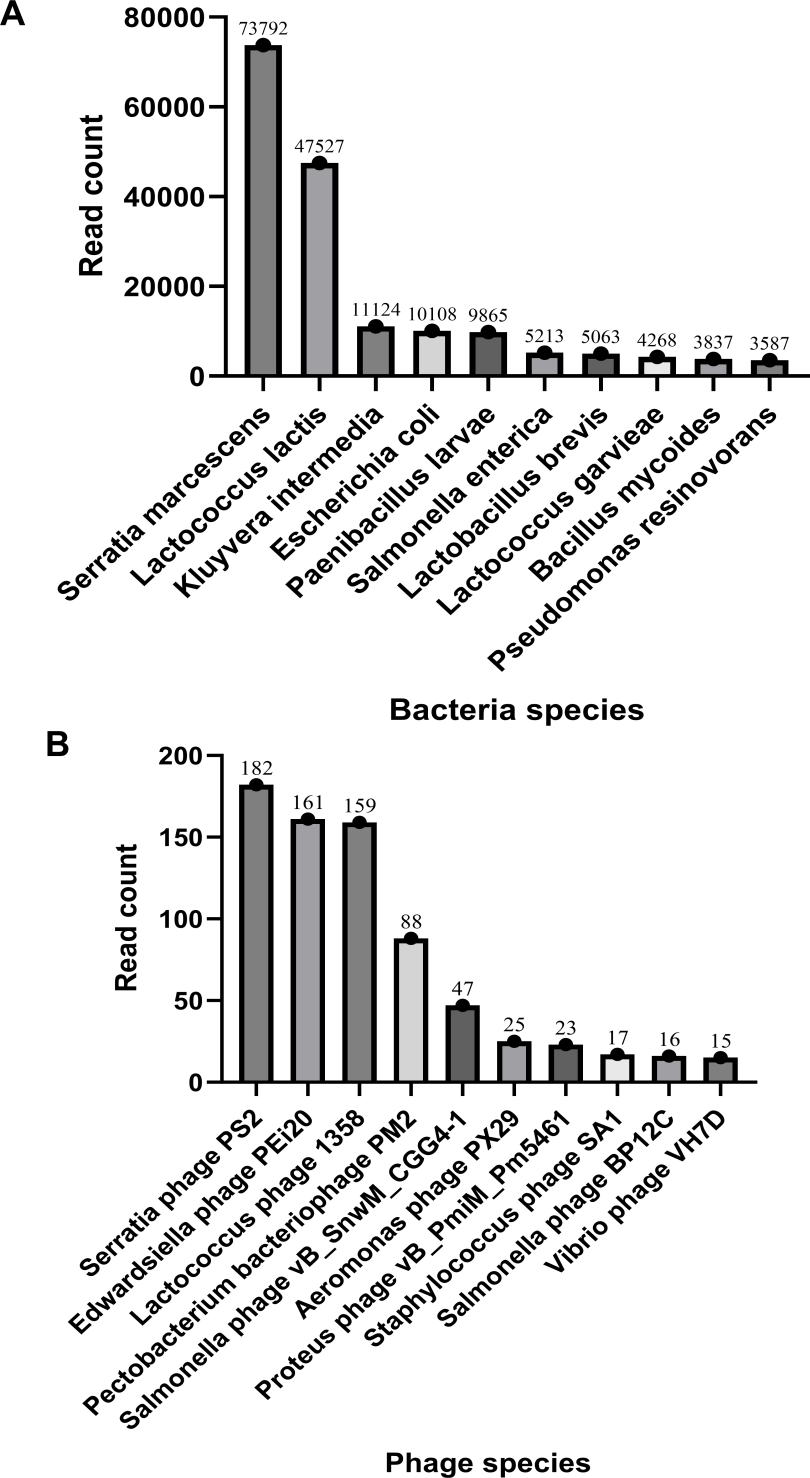
Taxonomic annotation of clean reads from the microbial DNA of the *P. americana* gut sample was conducted using Kraken2, and species abundance was further estimated with Bracken. (**A**) Bacteria with a higher number of reads are annotated as prokaryotic organisms. (**B**) Phages with a higher number of reads are annotated as viruses.

The clean sequencing reads were taxonomically classified and annotated using Kraken2, yielding the total read counts for both bacterial and viral sequences. To investigate the relationship between host bacteria and phages and identify the dominant phage species in the gut microbiota, we further employed Bracken to estimate the relative abundance of bacterial and phage species. Our analysis revealed that *S. marcescens* was the most abundant bacterial species in the gut (Fig. 1A), with 73,792 sequences, while *Serratia phage PS2* was the most prevalent phage in the viral community (Fig. 1B), represented by 182 sequences. The observed correlation between the abundance of *S. marcescens* and *Serratia phage PS2* suggests a potential close interaction between them, further supporting that *Serratia phage PS2* exhibits significantly higher abundance in the gut compared to other phages.

### Species abundance based on the sum of gene abundance

**Fig 2:**
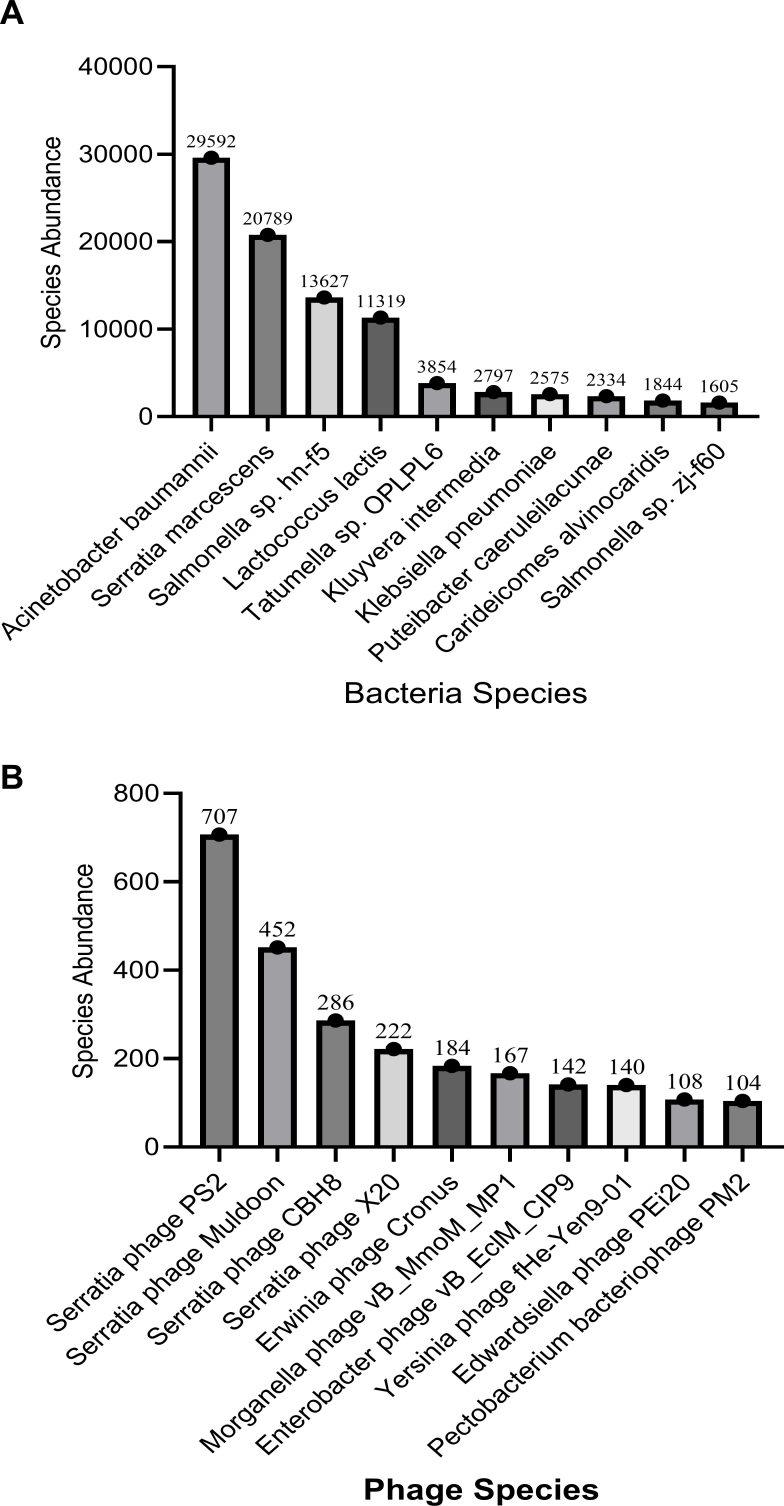
Bacterial and phage species abundance plot. (**A**) Species abundance plot in bacteria (Top 10). (**B**) Species abundance plot in phages (Top 10).

We constructed the species abundance profiles of bacteria and phages in the gut of *P. americana* by performing BLASTP alignment of the gene set obtained from contig assemblies against corresponding taxonomic databases for species annotation, followed by calculating species abundance based on the sum of gene abundances. In the bacterial species abundance profile (Fig. 2A), *Acinetobacter baumannii* (29,592) showed higher abundance than *S. marcescens* (20,789). This result exhibited some discrepancy with Fig. 1A, which may be attributed to the gene abundance analysis where certain sequencing reads could map to multiple highly similar genes (e.g., different genes of the same species or homologous genes across species), leading to read multi-counting and consequently inflated abundance estimates. Nevertheless, *S. marcescens* still maintained significant abundance in the *P. americana* gut. In the phage species abundance profile (Fig. 2B), we observed results consistent with Fig. 1B, with *Serratia phage PS2* demonstrating particularly prominent abundance. Based on the species abundance profiles, read taxonomic annotation, and potential host-microbe-phage interactions, we concluded that *S. marcescens* phages are substantially enriched in the *P. americana* gut. To validate this finding, we conducted subsequent experimental investigations.We have deposited the gut metagenomic data of *Periplaneta americana* in the SRA database under accession number PRJNA1269747.

### Isolation and morphological characterization of phage WSPA

**Fig 3:**
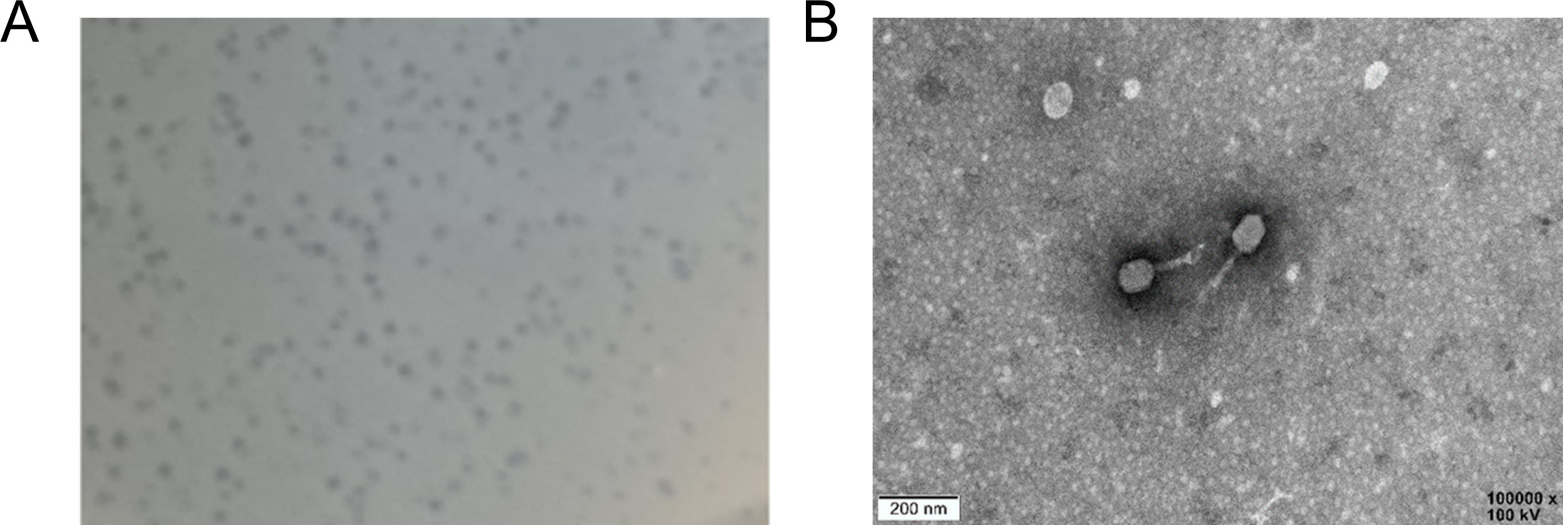
Phage plaques and phage virion morphology. (**A**) Plaques formed by the phage WSPA on the culture medium of the host bacterium *S. marcescens *18. (**B**) Electron micrograph of the phage WSPA, with arrows indicating the tails.

In three out of the four sets of samples collected from *P. americana*, plaque-forming units (PFU) were observed. A single isolate that produced the clearest and largest plaques was selected and purified. Phage WSPA was repeatedly purified and propagated on double-layer agar plates containing the host bacterium *S. marcescens *18, forming uniform plaques with a diameter of approximately 0.63 ± 0.06 mm, clear and transparent edges, and a phage titer reaching 1.08 × 10^9^ PFU/mL (Fig. 3A). This makes it suitable for further characterization and evaluation as a potential biocontrol agent. Based on morphological characteristics, tailed phages were classified (43), and electron microscopy revealed distinct head and tail structures with a total length of 225 ± 2 nm. The head had a diameter of approximately 110 ± 2 nm, featuring a prominent ortho-polyhedral structure, and the tail measured 115 ± 2 nm in length (Fig. 3B). The morphological observations indicated that phage WSPA exhibits a *Myovirus*-like morphology. Here, we describe only the morphological characteristics, and further classification will be based on the sections “Characterization of phage WSPA genome” and “Phylogenetic analysis.”

### Host range

The host range of phage WSPA was evaluated using the DLA method through spot testing. The host range analysis was performed on 47 bacterial strains, including *Shigella*, *Yersinia pseudotuberculosis*, *Yersinia enterocolitica*, *S. marcescens*, and other clinical bacterial genera (Table 3). The results indicated that, except for *S. marcescens* and one strain of *S. odorifera* within the *Serratia* genus, phage WSPA did not lyse any other bacterial genera. Among the 17 tested strains of *S. marcescens*, 14 were lysed, resulting in a lysis rate of 86.25%. This demonstrates that phage WSPA exhibits specificity toward the *Serratia* genus and is capable of targeting multiple strains within *S. marcescens*.

**TABLE 3:** Host range of the mucoid Serratia phage WSPA at different temperatures^*a*^

Species	Strain	21°C	28°C	37°C
*Serratia marcescens*	40	+	+	+
	43	+	+	+
	42	+	+	+
	41	+	+	+
	*Serratia odorifera*	+	+	+
	18=Ref	+	+	+
	20	+	+	+
	21	+	+	+
	29	+	+	+
	7	+	+	+
	45	+	+	−
	46	+	+	−
	47	−	−	−
	26	−	−	−
	44	−	−	−
	13	+	+	+
	14	+	+	−
Other clinical bacterial strains	*Pseudomonas aeruginosa*	−	−	−
	*Klebsiella pneumoniae*	−	−	−
	*Escherichia*	−	−	−
	*Acinetobacter baumannii*	−	−	−
	*Escherichia*	−	−	−
	*Salmonella typhi*	−	−	−
	*Enterobacter cloacae*	−	−	−
	*Buttiauxella agrestis*	−	−	−
	ATCC25922	−	−	−
	*Staphylococcus epidermidis*	−	−	−
*Shigella*	*Shigella flexneri* 1a	−	−	−
	*Shigella flexneri* 2a	−	−	−
	*Shigella flexneri* 3a	−	−	−
	*Shigella flexneri* 4a	−	−	−
	*Shigella flexneri* IV	−	−	−
	*Shigella flexneri* serotype X	−	−	−
	*Shigella sonnei* I	−	−	−
	*Shigella sonnei* II	−	−	−
	*Shigella dysenteriae* I	−	−	−
	*Shigella dysenteriae* II	−	−	−
	*Shigella boydii*	−	−	−
	*Shigella sonnei*	−	−	−
*Yersinia pseudotuberculosis*	PSTI	−	−	−
	PSTII	−	−	−
	PSTIII	−	−	−
	PSTIV	−	−	−
	PSTV	−	−	−
	PSTVI	−	−	−
*Yersinia enterocolitica*	52202	−	−	−
	52301	−	−	−

^*a*^ “Ref” indicates that the bacterial strain was used as a host strain. +, phage WSPA exhibits lytic activity; −, phage WSPA showed no lytic effect.

### Optimal multiplicity of infection determination

**Fig 4:**
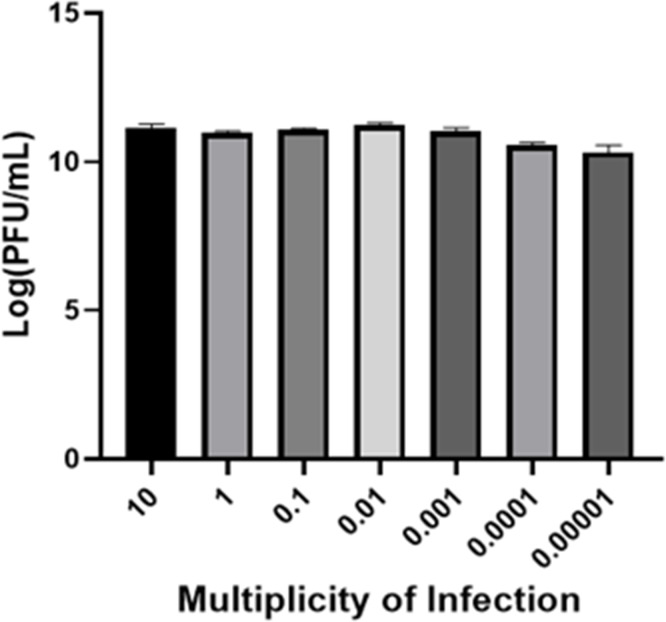
Optimal multiplicity of infection for the phage WSPA. The optimal multiplicity of infection for phage WSPA is represented as the mean ± standard deviation (SD). After 6 hours of cultivation, 10 µL is mixed with 100 µL of host bacteria and allowed to stand for 5 to 10 minutes, and the titer is determined by the DLA method.

In this study, the optimal MOI for phage WSPA was determined (Fig. 4). The results indicated no significant differences in phage titer across different infection ratios. Although the findings showed no significant variation in the proliferation efficiency of phage WSPA under different MOI conditions, based on the numerically higher progeny phage titer observed at an MOI of 0.01, we decided to select an MOI of 0.01 for subsequent experiments to further validate its potential effects.

### Biological properties of phage WSPA

**Fig 5:**
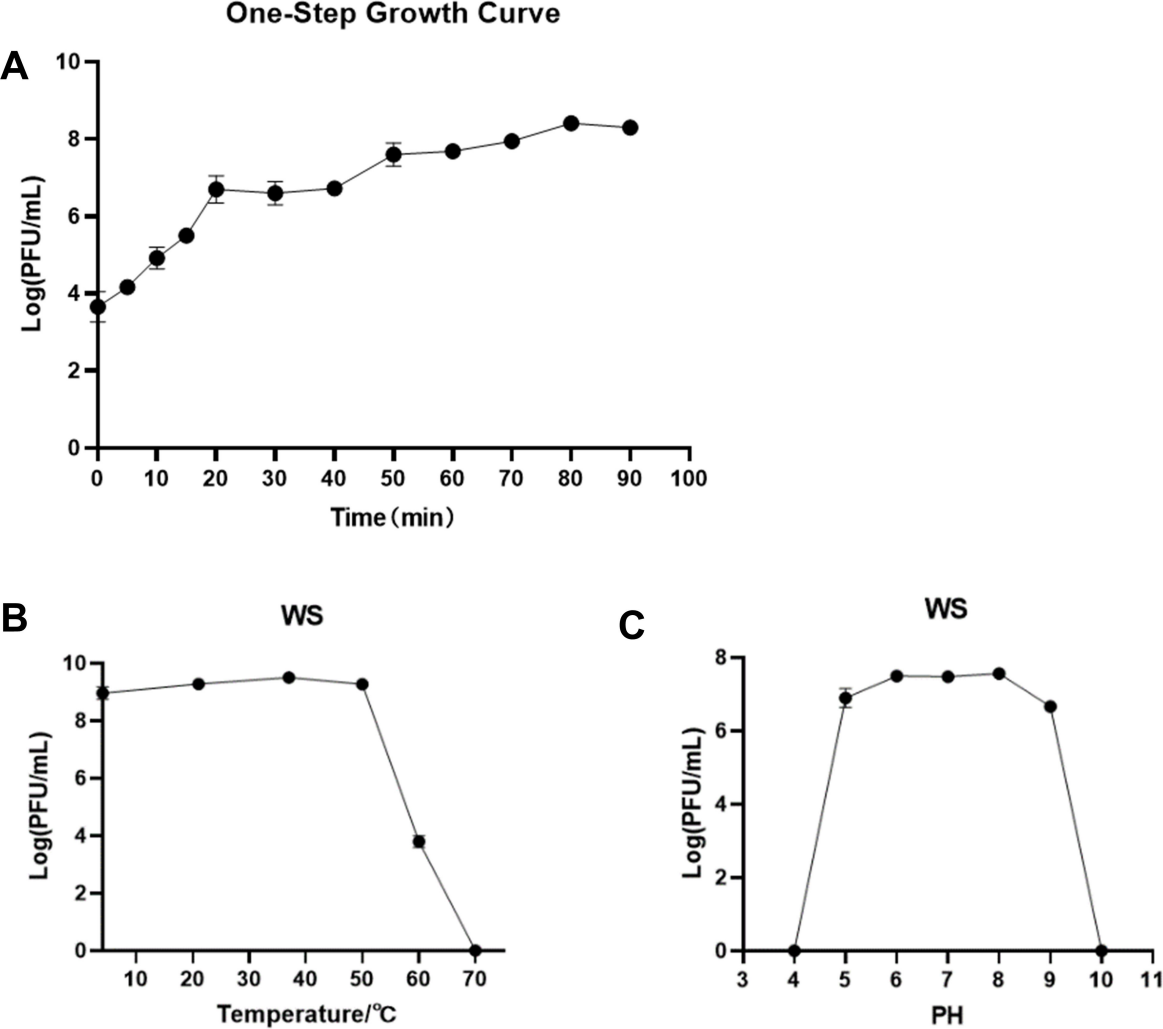
Biological characteristics of phage WSPA. (**A**) One-step growth curve of WSPA in *S. marcescens *18 at 37°C. The curve was determined in LB liquid medium at an MOI of 0.01 at 37°C. (**B**) Temperature tolerance after 1 hour treatment at 4°C, 21°C, 37°C, 50°C, 60°C, and 70°C. (**C**) pH stability of phage WSPA after 1 hour treatment under various pH conditions. Repeated three times. Data are reported as the mean ± standard deviation (SD).

The one-step growth curve of phage WSPA revealed no distinct latent phase during the initial observation period (Fig. 5A). The phage entered exponential growth within 20 minutes, with the burst size peaking at this time point. This rapid transition to exponential growth may have masked a potential latent period. This phenomenon could be attributed to the 5 minute sampling intervals during the first 20 minutes, where a latent period shorter than 5 minutes might have escaped detection. Notably, the phage titer reached a plateau between 20 and 40 minutes, followed by a slight rebound after 40 minutes, suggesting the initiation of secondary infection cycles. By 50 minutes, the titer stabilized at approximately 1 × 10⁸ PFU/mL, forming a second plateau. Although exponential growth characteristics were evident at 20 minutes, the subsequent plateau phase and minor titer fluctuations indicate that the one-step growth kinetics of phage WSPA may be more complex than those of other phage types.

To evaluate the thermal stability of phage WSPA, it was incubated at different temperatures for 1 hour, and the titer was measured (Fig. 5B). The results showed no statistically significant differences in phage titer among the 4°C, 21°C, 37°C, and 50°C treatment groups (*P* > 0.05), demonstrating good stability within this temperature range. However, when the temperature exceeded 50°C, the phage titer decreased significantly (*P* < 0.05) and was nearly completely inactivated at 70°C.

The stability of phage WSPA was examined across a pH range of 0–14 (Fig. 5C). The phage demonstrated high stability within the pH 6–8 range, showing no statistically significant differences in titer (*P* > 0.05). Partial inactivation with significant titer reduction (*P* < 0.05) was observed at pH 4–6 or 8–10. Complete inactivation (titer = 0) occurred under extreme pH conditions (pH 0–4 or 10–14).

### Characterization of phage WSPA genome

**Fig 6:**
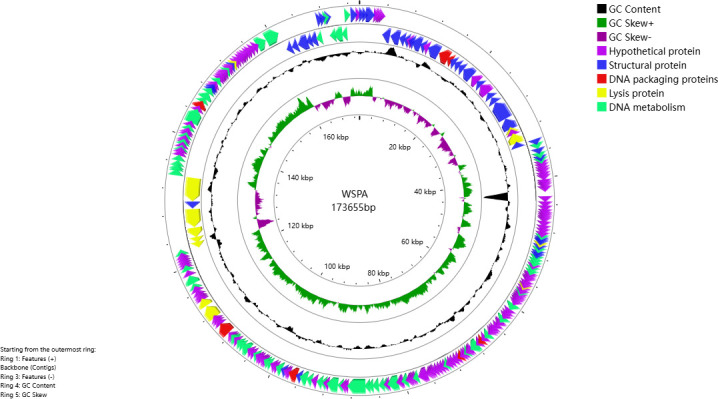
Genomic map of phage WSPA. The genomic structure of phage WSPA is represented in four circles. The first circle displays CDSs on the positive strand. The second circle displays CDSs on the negative strand. The third circle illustrates the GC content: outward peaks indicate regions where the GC content is higher than the average GC content of the entire genome, with taller peaks representing greater deviations; inward peaks indicate regions with lower GC content than the average, with taller peaks representing greater deviations. The fourth circle represents the GC skew value, calculated as (G − C)/(G + C); a positive value suggests a bias toward CDS transcription on the positive strand, while a negative value suggests a bias on the negative strand. The innermost circle displays the genome size.

**Fig 7:**
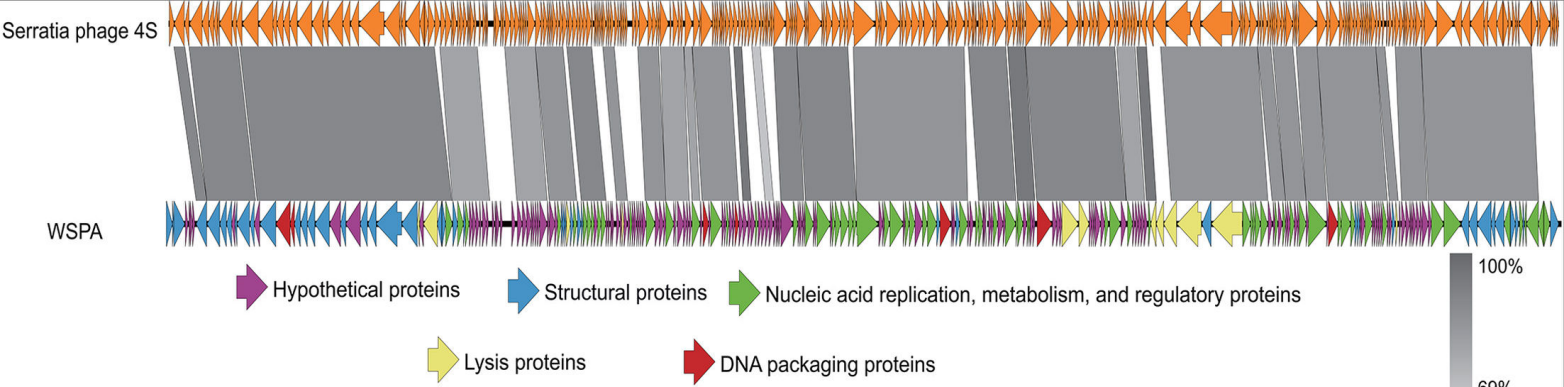
Multiple sequence alignment of phage genomes. Comparative analysis of the complete genomes of WSPA and *Serratia phage 4S* was performed using Easyfig. Gray-shaded regions indicate sequence similarities between the genomes.

The ICTV species demarcation criteria for bacterial and archaeal viruses primarily rely on genomic sequence similarity, with ≥95% identity required for strains to be considered the same species (44). Under this framework, analysis methods such as BLASTn stipulate that nucleotide sequence divergence within a species should not exceed 5%. Phage WSPA possesses a linear double-stranded DNA genome of 173,655 bp with a GC content of 40.09% (Table 4). tRNAscan analysis identified six tRNA genes, and the genome contains 273 ORFs, with 231 transcribed in the forward direction and 42 in the reverse direction (Fig. 6). Genome BLASTn alignment against all *S. phages* in the NCBI database revealed that WSPA shares only 86.92% sequence identity with its closest relative, *Serratia phage 4S* (GenBank accession: MW082584.1), with a query coverage of 86% (Table 5). Functional annotation through five major databases (NRDB, KEGG, COG, GO, and SWISS-PROT) assigned putative functions to 124 ORFs (45.42%), while the remaining ORFs were classified as hypothetical proteins. The multiple sequence alignment with *Serratia phage 4S* (Fig. 7) revealed sequence identity only in specific genomic regions, indicating significant divergence between the two genomes. Following the classification scheme established by Xi et al. (45), these encoded proteins were categorized into five functional groups based on sequence alignment: structural proteins, DNA packaging proteins, nucleic acid replication and metabolic regulation proteins, host lysis proteins, and hypothetical proteins.

**TABLE 4:** Summary of genome assembly results

Sample ID	WSPA
Total length	173,655
GC content (%)	40.09
N rate (%)	0.384
Type	Linear

**TABLE 5:** Partial results of genome BLASTn sequence identity analysis for phage WSPA

Scientific name	Percent identity (%)	Query coverage (%)
*Serratia phage 4S*	86.92	86
*Serratia phage PS2*	80.77	3
Serratia phage 92A1	75.70	44
Serratia phage Muldoon	70.57	32
Serratia phage SP1	70.20	31
Serratia phage vB SmaS Niamh	No significant similarity found	\^*a*^
Serratia phage vB SmaS Carrot	No significant similarity found	\
Serratia phage vB SmaS Swain	No significant similarity found	\
Serratia phage KpZh 1	No significant similarity found	\
Serratia phage MQ-4	No significant similarity found	\
Serratia phage vB SspM BZS1	No significant similarity found	\
Escherichia phage T7	No significant similarity found	\

^*a*^ The "\" symbol indicates no sequence identity was detected in the BLASTn alignment.

The phage DNA packaging machinery comprises the terminase large subunit (ORF18) and small subunit (ORF19), which are fundamentally involved in viral genome packaging. Specifically, the ORF18-encoded large subunit mediates DNA translocation, whereas the ORF19-encoded small subunit initiates the packaging process through molecular interactions with its large subunit counterpart (46). These proteins are ubiquitously distributed within the phage particle and play pivotal roles in its DNA packaging process.

The nucleic acid replication, metabolism, and regulatory protein module of phage WSPA contains multiple key proteins, including DNA primase (ORF161), DNA helicase (ORF174), DNA ligase (ORF257), and exonuclease A (ORF176). ORF161 and ORF174 show sequence identity to the primase and helicase of *Erwinia phage Cronus* and *Enterobacter phage PG7*, respectively. Given that DNA replication is semi-discontinuous, primase is required for the replication of the latter strand. These proteins bind to the N-terminus of helicase to create a replication fork, essential for DNA replication and transcription. Helicases and primases are essential for the complete unwinding of DNA during catalytic synthesis (47). Furthermore, ORF176 and ORF257 encode putative nuclease exonuclease and DNA ligase, respectively, assisting in DNA synthesis and regulating DNA replication, recombination, and repair. The presence of a complete set of functional proteins within this module suggests that phage WSPA has an independent DNA replication, metabolism, and packaging system.

The lysis module includes phage lysozyme murein hydrolase (ORF67), holin lysis mediator (ORF203), among others. Among these, the proteins encoded by ORF67 and ORF203 mediate host lysis—holin, as a membrane protein, regulates infection cycle duration to ensure optimal lysis, while lysozyme degrades bacterial cell wall peptidoglycan to disrupt the membrane. This module fully illustrates the phage’s lysis mechanism and its effects on the host bacterium. Notably, BLASTP analysis revealed that despite the presence of a genome assembly gap at position 42,361, both the lysozyme murein hydrolase and holin lysis mediator proteins still maintained high sequence homology with their corresponding counterparts in *Serratia phage 4S*, demonstrating amino acid sequence identities of 86.42% (E = 3e^−97^) and 99.54% (E = 2e^−159^), respectively, with both alignments achieving 100% query coverage (Table 6).

**TABLE 6:** The sequence identity between WSPA’s lysozyme murein hydrolase/holin lysis mediator and their corresponding proteins in *Serratia phage 4S*

Functional protein	Percent identity (%)	Query cover (%)	E value
Lysozyme murein hydrolase	86.42	100	E = 3e^−97^
Holin lysis mediator	99.54	100	E = 2e^−159^

Additionally, genomic analysis indicates that phage WSPA carries no identifiable lysogeny-associated genes, with no detectable antibiotic resistance or virulence genes identified through online database screening. Preliminary characterization indicates that this phage may function as an obligately lytic phage specifically to *S. marcescens*. While demonstrating potential therapeutic value for controlling nosocomial infections, these clinical applications require further experimental validation.

We have deposited the genome sequence of phage WSPA in GenBank under the accession number PQ600363.1.

### Phylogenetic analysis

**Fig 8:**
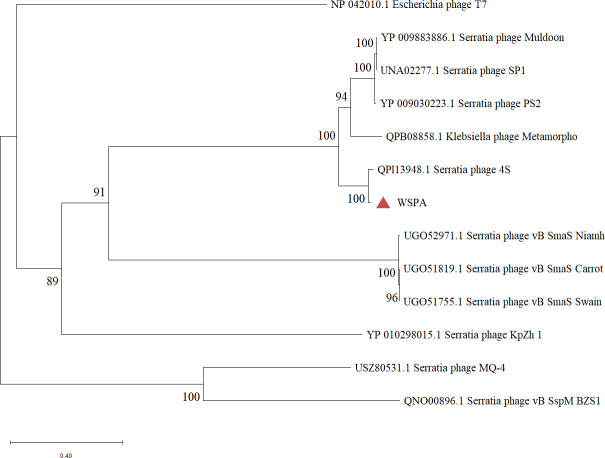
Perform a phylogenetic analysis of phages WSPA based on the terminase large subunit. Construct a phylogenetic tree using the neighbor-joining method (the percentage of replicate trees in which the associated taxa clustered together in the bootstrap test [1,000 replicates] is shown next to the branches [49]).

According to the latest guidelines from the ICTV, genus-level classification requires ≥70% genome average nucleotide identity (ANI) (48). Phylogenetic analysis based on the terminase large subunit sequence identity demonstrated that phage WSPA clusters with *Serratia phage 4S* on the same evolutionary branch, forming a distinct clade with five other *Muldoonvirus* phages (Fig. 8). ANI analysis conducted using the CJ Bioscience online platform (https://www.ezbiocloud.net/tools/ani) revealed 85.77% ANI between WSPA and *Serratia phage 4S* (Table 7), thereby confirming previous findings. The phylogenetic analysis further establishes that WSPA represents a novel phage within the genus *Muldoonvirus* of the class *Caudoviricete*s

**TABLE 7:** Phage WSPA and *Serratia phage 4S* OrthoANIu results

Metric	Value
OrthoANIu value (%)	85.77
Average aligned length (bp)	91,694
Genome WSPA coverage (%)	52.88
Genome *Serratia phage 4S* coverage (%)	53.19

## DISCUSSION

This study reports the isolation and characterization of a novel bacteriophage, WSPA, from the gut microbiome of *P. americana* (Fig. 3B). Phage WSPA, along with other *S. marcescens* phage*,* such as *Serratia phage 4S*, is classified as a T4-like phage. Genome analysis identified 124 ORFs with known functions, including most of the functional genes essential for phage reproduction. However, 149 ORFs did not correspond to any known functions in the NCBI database. Further functional studies on these ORFs will deepen the understanding of phage WSPA.

Through genome sequencing and comparative genomic analyses (Fig. 6 and 7), coupled with phylogenetic reconstruction based on the highly conserved terminase large subunit gene (Fig. 8), we demonstrate that phage WSPA belongs to the genus *Muldoonvirus* within the *Caudoviricetes* class (45, 50). Notably, although the NCBI database contains extensive genomic data from *S. marcescens* phages, WSPA exhibits only 86.92% genome sequence identity with its closest relative, *Serratia phage 4S*, significantly below the 95% species demarcation threshold—supporting its classification as a novel species (Table 5). Transmission electron microscopy further confirmed that WSPA possesses characteristic myoviral morphology, featuring an icosahedral capsid and contractile tail assembly (Fig. 3B). Genomic annotation identified multiple conserved phage functional genes involved in DNA replication, transcriptional regulation, and structural protein formation. Most significantly, comparative analysis revealed no substantial differences in the lysis module components (lysozyme, murein hydrolase, and holin lysis mediator genes) between WSPA and *Serratia phage 4S* (Table 6), suggesting potential conservation of lytic mechanisms and host interaction strategies between these phages.

The host range specificity of phage WSPA is primarily determined by its tail fiber protein, which recognizes and binds to specific receptor sites on host cell surfaces, thereby playing a crucial role in phage-host recognition and adsorption (51–54). GenBank annotation revealed that WSPA contains only one tail fiber protein (ORF205), which exhibits 84.68% amino acid sequence identity with 100% query coverage to its counterpart in *Serratia phage 4S*, suggesting these phages may have a host range restricted to *S. marcescens*. Our host range analysis results demonstrate that phage WSPA exhibits specificity toward *S. marcescens* and can effectively infect multiple distinct strains within this species (55).

The phage genome encodes enzymes such as endonucleases (ORF135) and polymerases (ORF145), which allow it to disrupt host DNA, replicate and recombine DNA independently, and complete transcription independently. The lysis module, responsible for breaking down the host cell wall, facilitates the release of progeny phages, playing a crucial role in the invasion of the host bacteria by the phage and the release of progeny phages.

The phage genome contains six tRNAs, which are likely involved in the transcription process and may enhance the infectivity and lytic ability of phage WSPA, indicating that this phage has robust lytic ability and can effectively lyse the host bacteria. Analyzing the functional genes associated with lytic enzymes may reveal the phage’s lytic mechanism, providing insights into how it efficiently destroys host cells and may have significance for the development of new antibacterial therapies.

The latent period is one of the critical parameters in phage therapy. Generally, phages with shorter latent periods demonstrate more significant advantages in bacterial lysis (56). In this study, we employed a sampling protocol with 5 minute intervals during the first 20 minutes (detailed in “Multiplicity of infection and one-step growth curve” above), which may have failed to detect latent periods shorter than 5 minutes. Our results indicate that this phage exhibits no observable latent period and can rapidly complete the processes of infection, replication, and progeny release (Fig. 5A). The optimal MOI for this phage was determined to be 0.01, and the phage titer remained relatively stable even at an extremely low MOI of 0.00001 (Fig. 4).

Furthermore, phage WSPA maintains a high titer at temperatures below 50°C (Fig. 5B) and remains active in the pH range of 5–9 (Fig. 5C), indicating its broad tolerance to heat and pH. Most importantly, the genome of phage WSPA appears to lack lysogenic genes or virulence genes. Lysogenic phages can integrate their genomes into the DNA of host bacteria, entering a dormant state, which may result in the acquisition of new genes (such as antibiotic resistance genes or virulence factors) by the host bacteria, thereby increasing the risks associated with therapy. Therefore, the absence of lysogenic genes in phage WSPA makes it more suitable for phage therapy. Although these characteristics are encouraging, further experimental validation is still required to comprehensively evaluate their safety for humans and the environment. With additional assessment, it has the potential to serve as an antibiotic alternative for hospital-acquired infections and fulfill its promise as an antimicrobial agent.

### Conclusion

This study employed metagenomic sequencing to analyze the phage diversity in the gut of *P. americana*, revealing a substantial abundance of *S. marcescens*-targeting phages. Ultimately, phage WSPA was successfully isolated from the intestinal samples of *P. americana*. Previous studies have predominantly isolated phages from natural environments like soil, with limited research on phage acquisition from intestinal sources. Therefore, this work establishes a novel approach for phage procurement.

In clinical practice, prolonged monotherapy using a single phage type may lead to phage resistance. The adoption of phage cocktail therapy can simultaneously broaden the antimicrobial spectrum and effectively mitigate resistance risks. Consequently, it is imperative to isolate more phages with specific targeting capabilities, which is crucial for enhancing the efficacy of clinical phage therapy.

Through comprehensive genomic and proteomic characterization, we identified phage WSPA as a new member of the genus *Muldoonvirus* within the class *Caudoviricetes*. Despite the extensive collection of *S. marcescens* phage genomes in the NCBI database, WSPA shares only 86.92% sequence identity with its closest relative. This research laid a strong foundation for creating a medicinal insect phage library, which could advance biopharmaceutical development and clinical applications.

## Data Availability

The data will be made available upon reasonable request. The data are publicly accessible under the following accession numbers: metagenomic data, PRJNA1269747; phage WSPA viral genome, PQ600363.1.
